# Accuracy of Critical Care Pain Observation Tool and Behavioral Pain Scale to assess pain in critically ill conscious and unconscious patients: prospective, observational study

**DOI:** 10.1186/s40560-016-0192-x

**Published:** 2016-11-07

**Authors:** Paolo Severgnini, Paolo Pelosi, Elena Contino, Elisa Serafinelli, Raffaele Novario, Maurizio Chiaranda

**Affiliations:** 1 Department of Biotechnologies and Sciences of Life, Intensive Care Unit–ASST Sette Laghi–Ospedale di Circolo Fondazione Macchi, University of Insubria, Viale Luigi Borri 57, 21100 Varese, Italy; 2 Largo R. Benzi 10, 16132 Genova, Italy

**Keywords:** Pain, Critical Care Pain Observation Tool, Behavioral Pain Scale, Critical ill patients, Intensive care unit, Pain management

## Abstract

**Background:**

Critically ill patients admitted to intensive care unit (ICU) may suffer from different painful stimuli, but the assessment of pain is difficult because most of them are almost sedated and unable to self-report. Thus, it is important to optimize evaluation of pain in these patients. The main aim of this study was to compare two commonly used scales for pain evaluation: Critical Care Pain Observation Tool (CPOT) and Behavioral Pain Scale (BPS), in both conscious and unconscious patients. Secondary aims were (1) to identifying the most relevant parameters to determine pain scales changes during nursing procedures, (2) to compare both pain scales with visual analog scale (VAS), and (3) to identify the best combination of scales for evaluation of pain in patients unable to communicate.

**Methods:**

In this observational study, 101 patients were evaluated for a total of 303 consecutive observations during 3 days after ICU admission. Measurements with both scales were obtained 1 min before, during, and 20 min after nursing procedures in both conscious (n.41) and unconscious (n.60) patients; furthermore, VAS was recorded when possible in conscious patients only. We calculated criterion and discriminant validity to both scales (Wilcoxon, Spearman rank correlation coefficients). The accuracy of individual scales was evaluated. The sensitivity and the specificity of CPOT and BPS scores were assessed. Kappa coefficients with the quadratic weight were used to reflect agreement between the two scales, and we calculated the effect size to identify the strength of a phenomenon.

**Results:**

CPOT and BPS showed a good criterion and discriminant validity (*p* < 0.0001). BPS was found to be more specific (91.7 %) than CPOT (70.8 %), but less sensitive (BPS 62.7 %, CPOT 76.5 %). COPT and BPS scores were significantly correlated with VAS (*p* < 0.0001). The combination of BPS and CPOT resulted in better sensitivity 80.4 %. Facial expression was the main parameter to determine pain scales changes effect size = 1.4.

**Conclusions:**

In critically ill mechanically ventilated patients, both CPOT and BPS can be used for assessment of pain intensity with different sensitivity and specificity. The combination of both BPS and CPOT might result in improved accuracy to detect pain compared to scales alone.

**Trial registration:**

NCT01669486

## Background

Pain management in critically ill patients is a complex process, but relevant to the clinical management. Pain is highly underestimated although it seems to be the patients’ worst memory in intensive care unit (ICU) [1, 2] even after 5 years from ICU discharge [3]. The perception of pain in ICU patients is mainly associated with respiratory therapy, positioning of nasogastric tube, venous and arterial catheters, and lack of mobilization [2]. However, patients are usually unable to self-report their pain due to sedative drugs and intubation, likely leading to its underestimation [4, 5]. Pain with agitation and delirium has been reported to negatively affect outcome of mechanically ventilated patients [6]. Thus, it is required to have valid and reliable methods to assess pain in unconscious patients to optimize treatment [7]. There is no one standard approach to evaluate pain in ICU, and the proposed tools actually used have several advantages and disadvantages. In conscious patients, self-report, i.e., visual analog scale (VAS) is the gold standard for pain assessment [8]. In unconscious patient, new methods have been developed to assess pain by using behavioral scales [9–11]. In unconscious patients, two scales have been proposed to assess pain in ICU patients: Behavioral Pain Scale (BPS) [12] and Critical Care Pain Observation Tool (CPOT) [13]. However, the potential superiority of each of them for assessment of pain in mechanically ventilated patients is not well established [14, 15]. The main difference between CPOT and BPS is the evaluation of body movements and muscle tension. We hypothesized that CPOT is more sensitive and accurate to assess pain compared to BPS in critically ill patients being specifically focused on muscular tension.

The aims of the present study were the following: (1) to compare CPOT and BPS separately, in conscious and unconscious critically ill mechanically ventilated patients; (2) to identify the most relevant parameters to determine pain scales changes during nursing procedures; (3) to compare both pain scales with VAS; and (4) to identify the best combination of scales for evaluation of pain in patients unable to communicate.

## Methods

This was a prospective, mono-centric study registered at ClinicalTrials.gov (ID NCT01669486). The study was conducted in the ICU at “Ospedale di Circolo Fondazione Macchi Varese”. The study was revising by the local Ethical Committee (protocol n.0003412), and informed consent was obtained from the relatives or patients according to local regulations.

The staff is made up of 12 doctors and 28 nurses for 12 beds in a general intensive care unit. The patients-nurse ratio is 2:1 by day and 3:1 by night. The medical staff is also completed by five physicians in training. The most active phase of nursing care was made every morning and every afternoon. The nurses performed maneuvres for taking care of hygiene, therapy administration, and continuous monitoring of vital parameters. They are graduated in nursing after 3 years at university school and 2 years of master in intensive care. The medical staff is provided by physician graduated in medicine and surgery and specialized in anesthesia and intensive care after 5 years in training. The medical staff during the study was responsible for the assessment of pain through the acquisition of the behavioral scale scores. Assessments have been carried out by medical staff trained to identify the presence of pain with two scales. These observations were not blinded. In the morning, nurses provide patients’ passive turning, cleaning, and repositioning; they perform airway suctioning, medications, and catheter management. In the afternoon, nurses provide only patient cleaning and repositioning. To get standardized measurements before, during, and after the maximum level of pain stimuli, we analyzed the behavioral scale scores during the nursing care performed in the morning [16].

The patients were evaluated with the Glasgow Coma Scale (GCS) and Sedation Agitation Scale (SAS). The conscious patients were identified with a GCS >10 and SAS = 4 (avoiding too agitated and too sedated patients to determine VAS).

Pain evaluation was performed in conscious and unconscious patients before, during, and 20 min after nursing care [17, 18]. Inclusion criteria were (1) need of invasive mechanical ventilation and (2) admission in ICU longer than 24 h. Exclusion criteria were (1) age <18 years old, (2) infusion of neuromuscular blocking agents, (3) any diseases causing tetraplegic and paraplegic condition as well as lateral neurological signs, and (4) pregnancy. After the enrollment day, every morning, the patients were evaluated for inclusion and exclusion criteria and, if feasible, we repeated the observations until tracheal extubation time, for a maximum of three observations for patient. We collected patients’ characteristics within 24 h after ICU admission, including age, gender, medical or surgical, SAS [19], and severity of illness by Acute Physiology and Chronic Health Evaluation (APACHE) and Simplified Acute Physiology Score (SAPS II). Pain assessment was performed by the CPOT and BPS scales in conscious and unconscious patients, while VAS in conscious patients only. In this study, the conscious patients were identified with a GCS greater than 10 and the patients that were able to answer with the VAS scale. The CPOT scale includes four behavioral indicators: facial expression, body movements, muscle tension, and compliance with the ventilator (Table 1). Each item is scored from 0 to 2 for a possible total score range from 0 to 8 points [15]. The BPS includes three behavioral indicators: facial expression, upper limb movements, and compliance with the ventilator (Table 1). Each item is scored from 1 to 4 for a possible score range from 3 and 12 points [12]. The VAS is a linear scale and identifies the pain by the self-report of patient, and it is considered the gold standard for evaluation of pain in conscious patients. In agreement with the literature [8], VAS ≥3 was used as a cutoff value to determine patients with pain. The combination of both BPS and CPOT scales was obtained by summing arithmetically the two scales normalized (Table 1). The two scales were normalized to convert the numeric scores from each scale. We compared CPOT subscale Body Movement and Muscle Tension with BPS subscale Upper Limb Movement and we summed the point of subscales. We considered a BPS score 3-4 and CPOT score 0-1-2 like absence pain; a BPS score 5-6-7 and CPOT score 3-4 like moderate pain; and a BPS score 8-9-10-11-12 and CPOT score 5-6-7-8 like severe pain.

**Table 1:** Behavioral Pain Scale, Critical Care Pain Observation Tool, Behavioral Pain Scale and Critical Care Pain Observation Tool combination

BPS score		CPOT score				BPS and CPOT combination score
Facial Expression		Facial Expression				
Relaxed	1	Relaxed, neutral		0		1
Partially Tightened	2	Tense		1		3
Fully tightened Grimacing	3 or 4	Grimacing		2		5 or 6
Upper Limb Movement		Body Movement		Muscle Tension		
No movement	1	Absence of movements	0	Relaxed	0	1
Partially bent	2	Protection	0 or 1	Tense, rigid	0 or 1	3 or 4
Fully bent with finger flexion Permanently retracted	3 or 4	Restlessness	0, 1, or 2	Very tense, or rigid	0, 1, or 2	5, 6, 7, or 8
Ventilator Compliance		Ventilator Compliance				
Tolerating movement	1	Tolerating ventilator		0		1
Coughing but tolerating for the most of time	2	Coughing but tolerating		1		3
Fighting ventilator Unable to control ventilation	3 or 4	Fighting ventilator		2		5 or 6

The table shows the Behavioral Pain Scale (BPS) (first column), the Critical Care Pain Observation Tool (CPOT) scores (second column), and the BPS and CPOT combination score (third column). The individual BPS and CPOT scores for each raw were summed. The BPS and CPOT combination score was obtained from the individual BPS and CPOT combination score from each raw. This combined BPS and CPOT score ranges from 3 to 20

### Statistical analysis

The statistical calculations were performed with in MedCalc for Windows, version 12.1.4.0. First, we calculate the sample size. The sample size calculation determined the number of the patients to enroll, and it was calculated a “priori” and based on 0.05 type I error and 0.20 type II error with a difference expected of 10 % order of magnitude in the area under the curve (AUC) area. We evaluated the validity to both scales, as criterion validity and discriminant validity. Discriminant validity refers to the ability of an instrument to measure the presence or the absence of the variable. In this study, the discriminant was correlated with the mean scores of both scales before and during the nursing care, 20 min after and during the procedure, and before and 20 min after the procedure, in conscious and unconscious patients. This correlation was calculated with Wilcoxon coefficient, which is a non-parametric test to determinate whether two samples come from the same statistical population, in the presence of ordinal values and continuous distribution. Box plot is a way to describe groups of numerical data through their quartiles. Criterion validity refers to the ability of an instrument to accurately measure the phenomenon of interest, in this case, the measurement of pain. It was evaluated by correlating the observed CPOT and BPS scores to the “gold standard” of pain measurement, i.e., VAS, when possible in conscious patients, and using the Spearman rank correlation coefficient (rs). Additionally, the sensitivity and the specificity of CPOT and BPS scores with ROC curve were assessed, only in conscious patients. The best discriminating between real and false positive is obtained by a curve passing in the upper left corner of at *x*/*Y* graphic (*x* = 1 − specificity, *y* = sensibility). In this case, the true positive corresponds to 100 % and the false positive correspond to 0 %. The AUC measures the ability of the scales to discriminate between patients who did or did not feel pain. The accuracy of individual scales was evaluated using calculation of sensitivity multiplied by the prevalence of positive to the sum of the observations (VAS ≥3, presence of pain) added to the specificity multiplied by the prevalence of negatives (VAS <3, absence of pain). Kappa coefficients with the quadratic weight were used to reflect agreement between the two scales [20], in conscious and unconscious patients. The Cohen’s Kappa is statistical coefficient that represents the degree of accuracy and reliability in a statistical classification; it is a concordance index calculated according to the ratio between the agreements in excess of the maximum obtainable [21]. The effect size is a quantitative measure of the strength of a phenomenon, for example, the correlation between two variables. We used this test to identify the most important subscale both BPS and CPOT, in conscious and unconscious patients. Cohen classified effect size as small (0.2–0.5), medium (0.5–0.8), large (0.8–1.3), and very large (>1.3) [22].

The level of significance accepted was a *P* value <0.05.

## Results

**Fig. 1:**
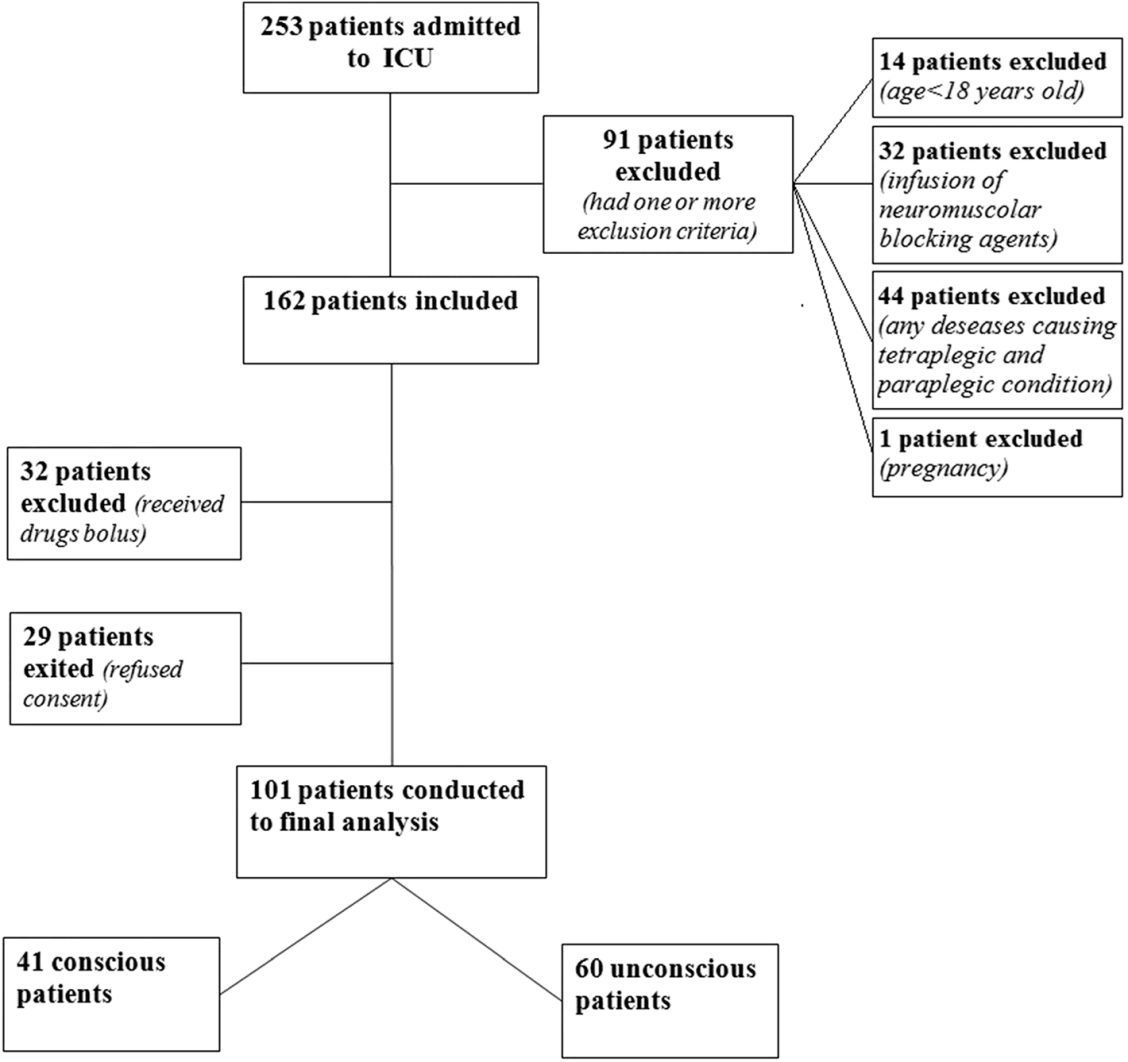
Consort flow diagram. Flow diagram summarizing inclusion, allocation, and analysis

In the study period (Fig. 1), 253 patients were admitted to the ICU and 162 patients met entry criteria. Among them, 61 patients were excluded (29 patients refused consent and 32 patients required sedation during the nursing care); thus, a total of 101 patients, 41 conscious and 60 unconscious patients, were included into the final analysis. In this study, the sample size was 75 patients/observations. In the enrollment phase, we needed to consider 101 patients to obtain 75 observations in conscious patients with VAS scale. The clinical characteristics of the patients are reported in Table 2. None patients presented delirium assessed through CAM-ICU scale. The analgesia and sedation of patients were obtained with the administration of midazolam (mean i.v. dose in unconscious patients was 0.04 ± 0.03 mg/kg/h, while in conscious patients 0.03 ± 0.028 mg/kg/h) and morphine (mean i.v. dose in unconscious patients 0.069 ± 0.12 mg/kg/h, while in conscious 0.061 ± 0.12 mg/kg/h) or propofol 2 % (mean i.v. dose in unconscious patients 0.14 ± 0.10 mg/kg/h, while in conscious patients 0.12 ± 0.10 mg/kg/h) and remifentanil (mean i.v. dose in unconscious patients 0.03 ± 0.01 mg/kg/h, while in conscious patients 0.02 ± 0.01 mg/kg/h) and they were kept constant during the procedure, minimizing any possible influence on the evaluation of pain. We interrupted the sedation to evaluate the level of consciousness of patients, when possible and useful, only after the nursing procedures and after the evaluation of the patients, not to interfere with the application of the scales.

**Table 2:** Clinical characteristics of the patients at the time of enrollment

Age (years)		65 ± 16.7
Gender (M/F)		64/37
APACHE II (mean ± SD)		15.7 ± 7.1
SAPS II (mean ± SD)		43.5 ± 13.4
SAS (mean ± SD)		3.0 ± 1.1
CAM ICU		(−) n: 101; (+) n: 0
Patient category	Medical (n)	33
	Surgical (n)	68
Outcome	ICU discharge (n)	88
	Died (n)	13

Data are expressed as mean ± standard deviation

*APACHE II* acute physiology and chronic health evaluation, *SAPS II* simplified acute physiology score, *SAS* sedation agitation scale, *CAM ICU* confusion assessment method for the intensive care unit; *ICU* intensive care unit, *(+)* presence, *(-)* absence, *(n)* number

The medical patients were affected to respiratory failure, from pulmonary edema and pneumonia. The surgical ones instead included patients undergoing abdominal, vascular, and thoracic surgery and multiple trauma. In addition to usual devices like central lines, arterial line, gastric tube, tracheal tube, and urinary catheter, these patients presented additionally surgical incisions, drainages, and open abdomen treatment.

We calculated discriminant validity for BPS and CPOT in overall, conscious and unconscious patients. BPS showed a statistically significant difference during nursing care (overall *Z* = −12.3, *p* < 0.0001; conscious *Z* = −6.93, *p* < 0.0001; unconscious *Z* = −10.68, *p* < 0.0001) and during and after nursing care (overall *Z* = −12.6, *p* < 0.0001; conscious *Z* = −6.78, *p* < 0.0001; unconscious *Z* = −11.15, *p* < 0.0001). We observed similar results with CPOT (during: overall *Z* = −12.09, *p* < 0.0001; conscious *Z* = −6.48, *p* < 0.0001; unconscious *Z* = −10.62, *p* < 0.0001; during and after, overall *Z* = −12.81, *p* < 0.0001; conscious *Z* = −6.64 *p* < 0.0001; unconscious *Z* = −11.36, *p* < 0.0001).

**Fig. 2:**
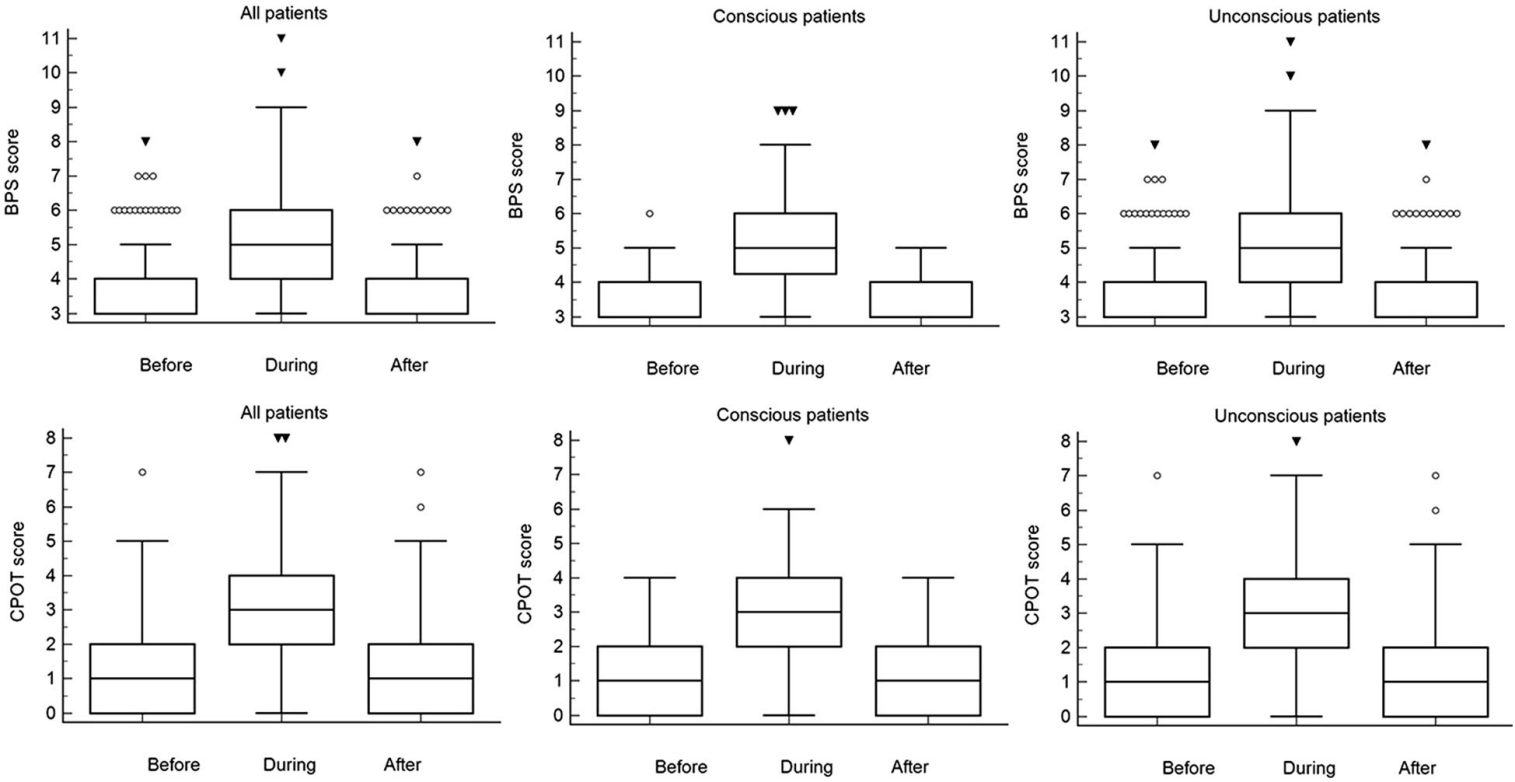
Variations BPS and CPOT values during the nursing. Variations Behavioral Pain Scale (BPS) and Critical Care Pain Observation Tool (CPOT) values in overall conscious and unconscious patients before, during, and after nursing procedures. *White column* identifies the box and whisker graph; the *vertical line* bar identifies results in three different moments. *Empty circles* and *triangles* show the maximum, minimum, and percentile values

Figure 2 shows changes in CPOT and BPS during nursing in overall, conscious and unconscious patients; both CPOT and BPS values increased during nursing while decreased at the end of procedure to come back to original status.

We compared the two scales in three different moments, with the Cohen’s Kappa, before *k* = 0.69, during = 0.64, after = 0.66; a k-value larger than 0.6 showed a good correlation.

Among different individual parameters for CPOT and BPS, in overall, conscious and unconscious patients, facial expression was the most important one for pain detection with effect size 1.4 while the other parameters presented an effect size more less (BPS scale Upper Limb Movement = 0.84, Ventilator Compliance = 0.99; CPOT scale Muscle Tone = 0.71, Body Movement = 0.60 and Ventilator Compliance = 1.09).

The criterion validity of BPS and CPOT scale showed a strong correlation with VAS, including all measurements (BPS rs = 0.56; *p* < 0.0001 CPOT rs = 0.48; *p* < 0.0001).

**Fig. 3:**
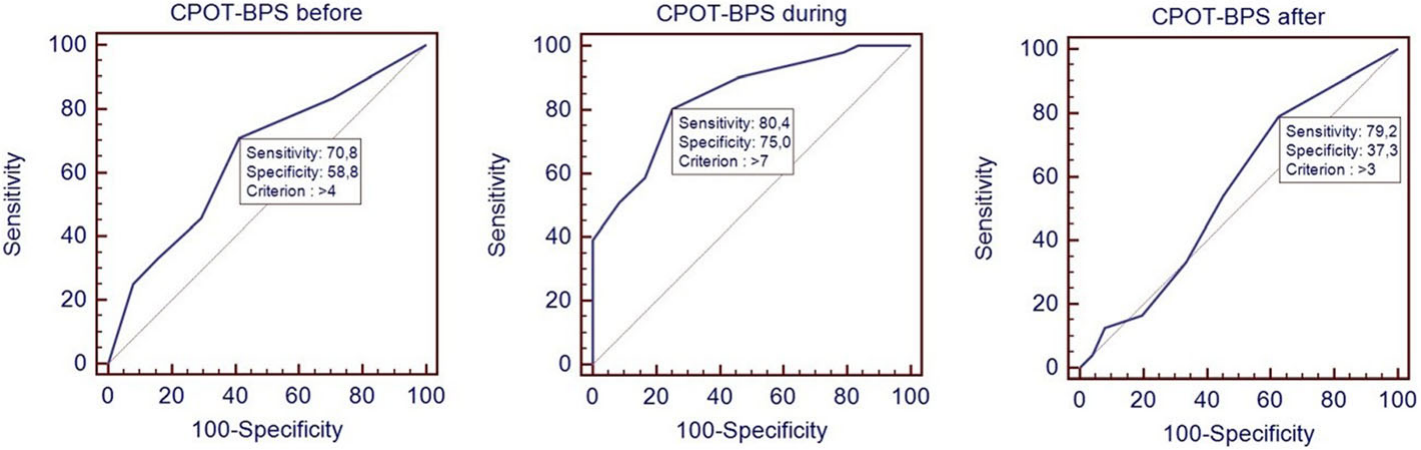
ROC curve of BPS and CPOT combination score. The curve identifies Behavioral Pain Scale (BPS) and Critical Care Pain Observation Tool (CPOT) combination score sensitivity and specificity compared with the gold standard Visual Analog Scale

Sensitivity and specificity to both scales and their combination are shown in Table 3 into three different moments. During the nursing care, in particular, we found a low sensitivity for BPS (BPS sensitivity 62.8 % and specificity 91.7 %, accuracy 72.04 %), and low specificity for CPOT (CPOT sensitivity 76.5 % and specificity 70.8 %, accuracy 74.68 %). The ROC curve obtained with the association of both BPS and CPOT scales, summing arithmetically the two scales normalized (Fig. 3), showed during the nursing specificity 75 % and sensitivity 80.4 %, with an accuracy 78.67 % with a AUC = 0.84.

**Table 3:** The table shows results of CPOT and BPS sensitivity and specificity

		Before	During	After	Overall	Cutoff value
BPS	Sensitivity	79.2	62.8	62.5	84.8	
	Specificity	61.2	91.7	60.8	52.3	5
	AUC	0.71	0.83	0.6	0.76	
CPOT	Sensitivity	25	76.5	33.3	48.5	
	Specificity	91.3	70.8	60.8	88.2	2
	AUC	0.57	0.8	0.5	0.7	
BPS and CPOT combination score	Sensitivity	70.8	80.4	79.2	50.5	
	Specificity	58.8	75	37.2	89	7
	AUC	0.65	0.84	0.55	0.74	

Sensitivity and specificity of BPS, CPOT as well as the association of both scales (BPS and CPOT combination score) compared to patient self-report before, during and after nursing maneuvers. Overall are all patients regardless of the stage of nursing, by entering into a single database all the measurements

*BPS* Behavioral Pain Scale, *CPOT* Critical Care Pain Observation Tool

## Discussion

In the present study, we evaluated two different pain scales in unconscious and conscious critically ill mechanically ventilated patients during nursing care. We found that (1) CPOT and BPS separately increased during nursing care in both unconscious and conscious patients and were significantly correlated; (2) facial expression showed greater changes for pain assessment; (3) in conscious patients, during nursing care, BPS showed higher specificity, and lower sensitivity compared to CPOT; and (4) the combination of both BPS and CPOT resulted in improved accuracy to detect pain compared to individual pain scales. Thus, against our expectations, our data suggest that CPOT was actually equivalent to BPS in sensitivity and accuracy for pain evaluation because none scale have better sensitivity and specificity to each other (BPS sensitivity 62.8 % and specificity 91.7 %, CPOT sensitivity 76.5 %, and specificity 70.8 %).

Our data suggest that using both BPS and CPOT during nursing care or other painful intervention might improve the evaluation of pain. To our knowledge, this is the first study suggesting that the combination of BPS and CPOT may be considered as a valuable tool for pain assessment in mechanically ventilated critically ill patients [15, 23, 24].

In the present study, we evaluated patients requiring mechanical ventilation and admitted to a general ICU. Thus, our results may be more easily applicable to a mixed population of critically ill patients. Few previous studies simultaneously recorded BPS and CPOT as well as VAS [15, 25, 26]. We compared both BPS and CPOT with VAS in conscious patients during nursing care, but our findings can be applied also to unconscious patients, being the pain assessment and the level of analgesia similar in both groups. These scales are normally used to evaluate pain in unconscious patients. Based on our own data, we demonstrated that BPS and CPOT might provide information about pain in unconscious patients, as shown by the correlation between VAS and BPS and CPOT. First, we showed a correlation between VAS and BPS and CPOT in conscious patients. Secondly, we obtained similar finding in unconscious patients.

Our results are in line with those reported in previous studies showing that both BPS and CPOT increase during nursing care and return back to baseline in a short period of time [27]. The diagnostic performance of both CPOT and BPS worsened after nursing care, suggesting that these scores might be affected by clinical maneuvres. Procedures like passive mobilization, i.e., turning and repositioning, and suctioning have been shown to increase pain. Conversely, active mobilization, i.e., rehabilitation, might be associated with less pain. In the present study, nursing procedures included turning and repositioning, but not routine suctioning and rehabilitation. Rehabilitation was not considered in the present study, since it might not induce pain due to active movements, better controlled by conscious patients only. Both BPS and CPOT have been evaluated in both unconscious and conscious patients. Pain assessment was similar in unconscious and conscious patients, suggesting that nursing care is painful independent of the level of sedation and analgesia [25]. Different individual items are included in BPS and CPOT. Muscular tone movement of arms and legs are included in CPOT but not BPS. Facial expression and ventilator compliance are recorded in both scales, although using different individual scores. We found that facial expression was the most important parameter related to pain assessment, in agreement with previous literature [15, 24]. It is important to note that facial expression is also easier to be scored at bedside. Furthermore, BPS and CPOT showed a good criterion and discriminant validity as previously reported [24–27], but BPS showed higher specificity but lower sensitivity compared to CPOT and so we cannot have considered CPOT superior to BPS, contrary to our hypothesis.

Thus, we hypothesized that of both BPS and CPOT, scales might result in improved accuracy to detect pain compared to each scale alone. The accuracy was evaluated by summing the scores of both scales for each individual observation. We found that during nursing care, the combination of BPS and CPOT resulted in better sensitivity. On the other hand, its specificity was higher than CPOT but lower than BPS. However, it was not possible to assess the best type of combination between the two scales, due to the higher prevalence of true positive cases and the limited sample size. In our study we did not find patients with delirium, and this may have been due to the optimization of the level of sedation.

Our study has limitations to be addressed. First, we evaluated only nursing care and no other possible painful maneuvers like suctioning. Second, the pain scales used are subjective to the operator and not objective. However, only one trained assessor evaluated pain for each patient. Third, we did not found any case of delirium in our analyzed patients, and this may be due to the level of sedative drugs applied, and/or to the feasibility of the CAM-ICU application in our population of critically ills patients.

Fourth, a relatively small group of patients was analyzed. Further studies are required to confirm our results in a larger population of patients.

## Conclusions

Both CPOT and BPS scales are applicable to detect pain in conscious and unconscious critically ill mechanically ventilated patients, but with different sensitivity and specificity. The association of both scales might improve the efficiency for pain assessment. The level of consciousness does not affect the perception of pain during nursing care.

## Abbreviations

APACHE: Acute Physiology and Chronic Health Evaluation
AUC: Area under the curve
BPS: Behavioral Pain Scale
CPOT: Critical Care Pain Observation Tool
ICU: Intensive care unit
rs: Spearman score
SAPS II: Simplified Acute Physiology Score
SAS: Sedation Agitation Scale
SD: Standard deviation
VAS: Visual analog scale
*Z*: Wilcoxon score
